# Measuring the tail: Methods for poly(A) tail profiling

**DOI:** 10.1002/wrna.1737

**Published:** 2022-05-26

**Authors:** Aleksandra Brouze, Paweł Szczepan Krawczyk, Andrzej Dziembowski, Seweryn Mroczek

**Affiliations:** ^1^ Institute of Genetics and Biotechnology, Faculty of Biology University of Warsaw Warsaw Poland; ^2^ Laboratory of RNA Biology International Institute of Molecular and Cell Biology Warsaw Poland; ^3^ Department of Embryology, Faculty of Biology University of Warsaw Warsaw Poland

**Keywords:** 3′‐end tailing, poly(A) tail, regulation of gene expression, RNA, RNA sequencing

## Abstract

The 3′‐end poly(A) tail is an important and potent feature of most mRNA molecules that affects mRNA fate and translation efficiency. Polyadenylation is a posttranscriptional process that occurs in the nucleus by canonical poly(A) polymerases (PAPs). In some specific instances, the poly(A) tail can also be extended in the cytoplasm by noncanonical poly(A) polymerases (ncPAPs). This epitranscriptomic regulation of mRNA recently became one of the most interesting aspects in the field. Advances in RNA sequencing technologies and software development have allowed the precise measurement of poly(A) tails, identification of new ncPAPs, expansion of the function of known enzymes, discovery and a better understanding of the physiological role of tail heterogeneity, and recognition of a correlation between tail length and RNA translatability. Here, we summarize the development of polyadenylation research methods, including classic low‐throughput approaches, Illumina‐based genome‐wide analysis, and advanced state‐of‐art techniques that utilize long‐read third‐generation sequencing with Pacific Biosciences and Oxford Nanopore Technologies platforms. A boost in technical opportunities over recent decades has allowed a better understanding of the regulation of gene expression at the mRNA level.

This article is categorized under:

RNA Methods > RNA Analyses In Vitro and In Silico

## Graphical abstract

A development of the methods used to study the length and composition of poly(A) tails, uncovering the 3′‐end tailing as a much more complex and impactful process than previously thought.

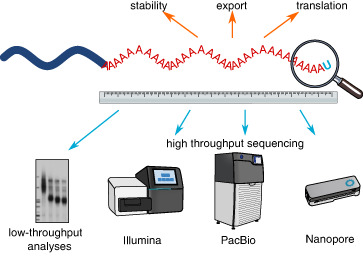

## 1 INTRODUCTION

Polyadenylation is the process by which nontemplated adenosines are added to the 3′‐end of mRNA, which occurs posttranscriptionally in the nucleus and is performed by canonical poly(A) polymerases. This modification affects almost all mRNA molecules except replication‐dependent histone mRNA. The poly(A) tail is necessary for the transport of mRNA molecules from the nucleus to the cytoplasm (Fuke & Ohno, 2008) and enhances mRNA stability, as deadenylation is the first step in RNA decay pathways (Wang & Kiledjian, 2001) and translatability through an interaction between poly(A)‐binding protein 1 (PABPC) to the 5′ cap‐binding translation initiation factor eukaryotic translation initiation factor eIF4G (Wakiyama et al., 2000; Wells et al., 1998; Xiang & Bartel, 2021).

**FIGURE 1:**
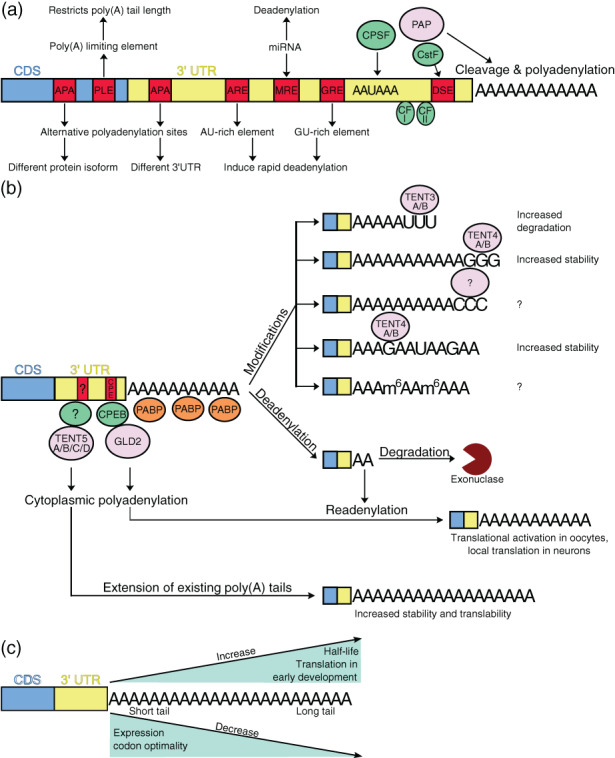
The poly(A) tail is a dynamic feature of mRNA. (a) *Cis*‐ and *trans‐*regulatory elements affect the poly(A) tail. (b) Possible fates of mRNA poly(A) tail in the cytoplasm. (c) Relationship between poly(A) tail length and different aspects of gene expression.

It is now commonly known that poly(A) tails are not merely unchanging, stable features of the 3′‐end of mRNA. The processes of polyadenylation, the regulation of poly(A) tail length, and its dynamic changes and modifications are controlled by a multitude of *cis*‐ and *trans*‐regulatory mechanisms and enzymes (Figure 1). Consequently, 3′‐end tailing is a much more complex and impactful process than previously thought.

Nuclear polyadenylation is preceded by endonucleolytic cleavage of the 3′‐end of mRNA with signals that designate the cleavage site within the transcript body between the highly conserved AAUAAAA hexamer and a U‐ or GU‐rich downstream sequence element (DSE). These elements serve as binding platforms for cleavage and polyadenylation specificity factor (CPSF) and cleavage specificity factor (CstF), respectively. After the recruitment of all necessary factors, including CPSF, CstF, cleavage factor I (CFI), CFII, and poly(A) polymerase, the cleavage of pre‐mRNA and finally, 3′‐end tailing occurs (Figure 1). Nascent homopolymers are then bound by poly(A) binding proteins (PABPs, PABPN1 in higher species; Proudfoot et al., 2002; Wahle & Rüegsegger, 1999).

However, more than half of human genes possess multiple polyadenylation sites (Tian et al., 2005). The usage of an alternative polyadenylation site (APA), constitutes an important regulatory mechanism. It can affect either the coding sequence, giving rise to different mRNA isoforms, or the 3′‐untranslated region (UTR), which influences mRNA fate. Sequences within the 3′‐UTR facilitate the binding of miRNAs or RNA binding proteins (Di Giammartino et al., 2011; Tian & Manley, 2016). One of the best known examples of alternative polyadenylation is the switch from the distal to proximal poly(A) site in mRNA that encodes immunoglobulin M (IgM), resulting in a transition from the membrane‐bound to secreted form of this protein during B cells differentiation to plasma cells (Alt et al., 1980; Early et al., 1980).

The choice of polyadenylation site and nuclear regulation of the mRNA poly(A) tail length depends on regulatory sequences within the transcript body. The presence of poly(A) limiting element (PLE) in the 5′ end of the terminal exon results in short poly(A) tails in albumin (Gupta et al., 1998) and transferrin (Gu et al., 1999) mRNAs in Xenopus that do not affect their decay rate. It also increases the efficiency of 3′‐end processing (Peng et al., 2005). AU‐rich elements (AREs) and GU‐rich elements (GREs) in the 3′‐UTR are linked with faster deadenylation and shortening of the poly(A) tail, leading to transcript degradation (C. Y. Chen et al., 2001; Vlasova et al., 2008; Xu et al., 1997). Similarly, miRNA binding to miRNA regulatory elements (MREs) in the 3′‐UTR induces the deadenylation and degradation of mRNA (L. Wu et al., 2006), playing an important role in the clearance of maternal transcripts during zebrafish early development (Giraldez et al., 2006). Interestingly, miRNA‐ and ARE‐mediated deadenylation can be connected. Dicer, a major component of miRNA processing machinery, is necessary for ARE‐mediated mRNA degradation, probably due to the requirement of miRNA for ARE‐RNA degradation. Indeed, miR16 targeted by ARE‐binding protein tristetraprolin (TTP) is necessary for ARE‐RNA turnover (Jing et al., 2005). Additionally, miRNA can interact with RNA‐binding proteins recognizing nearby motifs by regulating their activity (like Cpeb1), or conversely, RBP can either enhance miRNA activity or inhibits it (like HuR; Ciafrè & Galardi, 2013).

The length of the poly(A) tail and its dynamic changes play a crucial role in the life of the mRNA molecule (Figure 1b). It must be sufficiently long to facilitate mRNA export to the cytoplasm (Dower et al., 2004; Tudek et al., 2018). During the lifetime of mRNA, the poly(A) tail shortens. The bulk mRNA degradation pathway in eukaryotic cells begins with tail shortening mainly by CCR4‐NOT and PAN2/PAN3 deadenylases. Besides “canonical” poly(A) polymerases involved in cleavage‐dependent polyadenylation of nascent transcripts in the nucleus, the human genome encodes 11 “noncanonical” poly(A) polymerases (ncPAPs) and poly(U) polymerases (PUPs). Contrary to the canonical poly(A) polymerases, they are involved in extending the already existing poly(A) tail independently of transcript cleavage, they vary in nucleotide and substrate specificity, and some of these enzymes reside in the cytoplasm (Figure 1b; Liudkovska & Dziembowski, 2021). In oocytes and during early embryonic development, a fraction of deadenylated transcripts is not degraded but rather stored in a translationally inactive state (Barnard et al., 2004; J. H. Kim & Richter, 2006). After appropriate stimulation, Gld2 poly(A) polymerase catalyzes re‐adenylation and consequently the translational activation of these transcripts. This process is orchestrated by cytoplasmic polyadenylation element‐binding protein (CPEB), which recognizes a cytoplasmic polyadenylation element (CPE) that is within the 3′‐UTR of these transcripts (Richter, 2007; Villalba et al., 2011; Weill et al., 2012). In other cases, the extension of already existing poly(A) tails in the cytoplasm can increase stabilization and translation, such as in the case of immunoglobins or collagen transcripts in B cells (Bilska et al., 2020) and osteoblasts (Gewartowska et al., 2021), respectively. Tail elongation is catalyzed by proteins from the terminal nucleotidyltransferase 5 (TENT5) family of poly(A) polymerases, but the regulatory elements that drive this process remain unknown.

Finally, the poly(A) tail does not need to be composed only of adenosines. Recent studies revealed that 3′ ends of poly(A) tails are commonly uridylated (resulting in preferentially short poly[A] tails), guanylated (resulting in preferentially long poly[A] tails), or less frequently cytidilated (Chang et al., 2014), having diverse effects on mRNA stability. Uridylation by TENT3A and TENT3B (terminal uridylyltransferase 4 [TUT4] and TUT7) marks RNA for degradation, whereas guanylation by TENT4A/B protects mRNA from deadenylation, consequently increasing its stability (Lim et al., 2018). These modifications were also detected in the tail body (Y. Liu, Nie, Liu, & Lu, 2019b). Recently, a study of *Trypanosoma brucei* revealed the presence of methylated adenosines (m6A) within the poly(A) tail that protected them from deadenylation and stabilized the mRNA molecule (Viegas et al., 2022), thus revealing a new mechanism of the regulation of gene expression.

Poly(A) tails of steady‐state mRNAs are shorter than previously assumed (Chang et al., 2014; Lima et al., 2017; Subtelny et al., 2014). Highly expressed transcripts with high codon optimality possess relatively short poly(A) tails (Lima et al., 2017). Poly(A) tail length correlates positively with half‐lives, while with translation efficiency only in oocytes and early embryonic development (Chang et al., 2014; Lim et al., 2016; Subtelny et al., 2014; Figure 1c). Rapidly increasing interest in this area of research demands precise and high‐throughput tools to dissect and understand the nature and dynamics of mRNA poly(A) tail lengths. Historically, the first approaches that were based on RNaseH/oligo(dT) cleavage, followed by Northern blot analysis or the polymerase chain reaction (PCR)‐based amplification of poly(A) tails, provided information on only single transcripts and allowed only the observation of polyadenylation shift rather than subtle changes. These methods also did not provide any information about poly(A) tail length. The development of high‐throughput approaches, such as next‐generation sequencing (NGS), opened new possibilities for polyadenylation studies. The most common current approaches are based on the sequencing of appropriately prepared mRNA 3′ end libraries on an Illumina platform, with nucleotide‐level resolution. However, these estimations can be questionable because long homopolymer amplification is challenging, and PCR bias‐prone short‐read technology deprives us of information about very long poly(A) tails. Thus, single‐molecule, long‐read sequencing with Pacific BioSciences (PacBio) or Oxford Nanopore Technologies (ONT) platforms is recently gaining versatility and enabling non‐PCR‐biased poly(A) tail length estimations that can be accompanied by information about differential expression, splicing, and RNA modifications.

The present review provides details of the broad range of methods that are used for mRNA 3′‐terminome analyses, from the single‐transcript level to transcriptome‐wide studies that utilize second‐ and third‐generation sequencing.

## 2 THE BEGINNINGS OF POLY(A) TAIL LENGTH ESTIMATION

**FIGURE 2:**
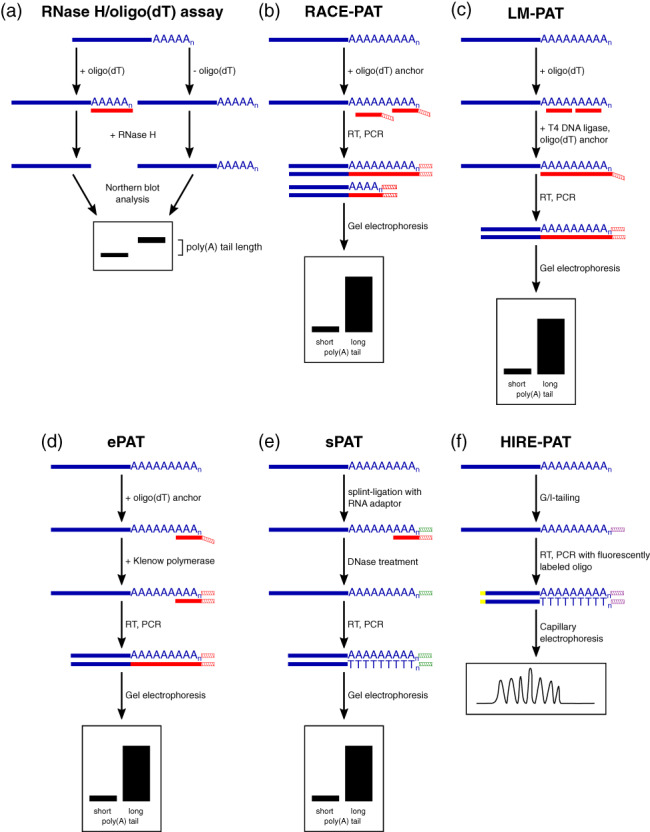
Overview of classic methods used to study poly(A) tail length ‐ (a) RNase H/oligo(dT) assay, and variations of PCR‐based Poly(A) Test (b) RACE‐PAT, (c) LM‐PAT, (d) ePAT, (e) sPAT, (f) HIRE‐PAT. The red line indicates the oligo(dT) sequence. Red hatched lines indicate the DNA 30 adapter sequence. Green hatched lines indicate the RNA 30 adapter sequence. Violet hatched lines indicate the G/I tag sequence. The yellow line indicates a fluorescent tag.

The first method that was applied to study poly(A) tail length was the RNase H/oligo(dT) assay, originally designed based on rabbit globin mRNA (Sippel et al., 1974). RNase H specifically hydrolyzes phosphodiester bonds in RNA only in RNA:DNA hybrids (Sippel et al., 1974). Thus, oligo(dT) hybridization to the poly(A) tail results in the recognition of this duplex by RNase H and subsequent removal of the poly(A) tail (Murray & Schoenberg, 2008). The length of poly(A) tails is assessed by Northern blot by comparing samples that are treated with RNase H in the presence or absence of oligo(dT) (Figure 2a). The difference in electrophoretic mobility reflects poly(A) tail length (Murray & Schoenberg, 2008; Sallés et al., 1999). However, differences in poly(A) tail length in the case of larger mRNAs (>2 kb) can be difficult to detect. Thus, to enable higher resolution, RNA is hybridized, along with oligo(dT), with a second antisense deoxyoligonucleotide near the 3′ end to trim mRNA (Murray & Schoenberg, 2008; Sallés et al., 1999).

Another approach to study poly(A) tails is the PCR amplification of 3′ ends. Classic PCR‐based assays for determining poly(A) tail lengths are the rapid amplification of cDNA ends poly(A) test (RACE‐PAT; also called the poly[A] length assay or PCR poly[A] test; Salles et al., 1992; Sallés et al., 1999) and ligase‐mediated poly(A) test (LM‐PAT). In RACE‐PAT, total RNA is incubated with an oligo(dT) anchor with a GC‐rich primer sequence on its 5′ end. It hybridizes along with the entire poly(A) tail length, eliciting heterogeneous cDNA populations. RNA is reverse transcribed from an oligo(dT) anchor primer, and the poly(A) tail of the gene of interest is PCR amplified with gene‐specific and oligo(dT) anchor primers (Figure 2b). The PCR products are analyzed by gel electrophoresis. Short poly(A) tails generate a band. In the case of longer poly(A) tails, amplicons with diverse poly(A) tail lengths create a smear on the gel (Murray & Schoenberg, 2008; Sallés et al., 1999). The LM‐PAT (Sallés & Strickland, 1995) differs from RACE‐PAT only in the oligo(dT) hybridization step design. Total RNA is hybridized with 5′‐phosphorylated oligo(dT) molecules in the presence of T4 DNA ligase, resulting in full coverage of the poly(A) tail. Therefore, the oligo(dT) anchor ligates to the 5′ end of the 3′‐most oligo(dT) (Figure 2c; Murray & Schoenberg, 2008; F. J. Sallés & Strickland, 1999; Sallés et al., 1999). Comparing these two methods, LM‐PAT is more sensitive to subtle changes in poly(A) tail length (Sallés et al., 1999). Both the RNase H‐ and PCR‐based approaches have advantages and limitations. Polymerase chain reaction‐based methods are principally faster, less laborious, and require a smaller amount of starting material than the RNase H assay. They are also relatively insensitive to internal poly(A) priming (annealing of oligo(dT) primer to poly(A) stretch within transcript body). However, the DNA polymerase is unable to amplify the exact number of nucleotides in a long homopolymer stretch in each template molecule, mostly shortening the homopolymer length and generating products with a wide range of sizes (PCR bias), whereas the RNase H method provides direct information about RNA without reverse transcription (RT)‐ or PCR bias.

Modifications of the classic PAT assay are based on different primers that are anchored to the 3′ end of RNA. The extension poly(A) test (ePAT; Jänicke et al., 2012) is based on the extension of the 3′ end of RNA by Klenow polymerase on a DNA template (Figure 2d). The splint‐mediated PAT (sPAT; Minasaki et al., 2014) utilizes splint ligation of the 3′ end of mRNA with an RNA anchor (Figure 2e). This approach is more sensitive than ePAT, requires less starting material and fewer amplification cycles, and is less biased toward shorter poly(A) tails (Minasaki et al., 2014). Other variations of the PAT assay include enzymatic G‐tailing (Kusov et al., 2001) or G/I‐tailing (Bazzini et al., 2012) of the 3′ end. The added tag serves as an anchor for a primer that consists of a C stretch followed by several Ts that are used for cDNA synthesis and PCR, accompanied by a gene‐specific primer (Kusov et al., 2001). Moreover, the fluorescent labeling of one of the primers allows for separation of the PCR product by capillary electrophoresis, resulting in very high resolution, referred to as the high‐resolution poly(A) test (Hire‐PAT; Bazzini et al., 2012; Figure 2f).

Different but less frequently used is a PCR‐based approach called circular RT‐PCR (cRT‐PCR; Couttet et al., 1997). Total RNA is decapped, which allows for RNA circularization by 5′‐ and 3′‐end ligation. After RT, nested PCR is performed to obtain products that cover the 3′–5′ junction, including poly(A) tails. The PCR products are visualized by gel electrophoresis in the presence of samples that are deadenylated at the beginning with RNase H to determine the poly(A) tail length (Couttet et al., 1997; Suh et al., 2006).

In conclusion, these methods contributed significantly to studies of poly(A) tails, thus opening a new, broad, and fascinating area of research. Although they are still commonly used for the validation of novel methods, single‐transcript analyses, and pilot experiments, they have many limitations and restricted applications. They only allow single‐transcript analyses of the poly(A) tail length shift, providing no precise poly(A) tail length or composition information. Consequently, the need for more accurate and higher throughput analyses led to the development of novel methods.

## 3 FIRST POLYADENYLATION STUDIES AT THE TRANSCRIPTOME‐WIDE SCALE WITH MICROARRAYS

The poly(A) tail is a distinctive feature of RNA that can be exploited for length‐based separation. Palatnik et al. (1979) demonstrated RNA fractionation based on the difference in poly(A) tail length using poly(U) chromatography, followed by the thermal elution of RNA. Alternatively, oligo(dT) chromatography and elution with decreasing salt concentrations can be applied (Jacobson, 1987). Such approaches were initially used only with single‐transcript analyses (Jacobson, 1987; Palatnik et al., 1979), but the development of microarray technology‐facilitated analyses of polyadenylation status at the transcriptome‐wide scale. RNA that is bound to biotinylated oligo(dT) and enriched on paramagnetic beads is eluted by buffers at decreasing salt concentrations. The polyadenylated and oligoadenylated fractions are then analyzed by microarrays (Meijer et al., 2007; Meijer & de Moor, 2011) to identify transcripts in each fraction. This method was used to show the polyadenylation of transcripts encoding cyclin B1, c‐mos, and Aurora‐A kinases during oocyte maturation in *Xenopus laevis* and in the mouse NIH3T3 fibroblast cell line (Meijer et al., 2007) and to study the effects of transcription dynamics on poly(A) tails (Slobodin et al., 2020). However, such a method deprives RNA with poly(A) tails that are shorter than 25 nt, whereas the abundance of short poly(A) tail transcripts constitutes 25% of expressed genes (Meijer et al., 2007). A comparative analysis of mRNA that was purified with cap‐binding eIF4E protein and oligo(dT) revealed a population of transcripts that were purified only or preferentially using the cap with poly(A) tails that were shorter than 30 nt (Choi & Hagedorn, 2003).

Poly(U) chromatography with thermal elution coupled with microarray analysis was used to study CPEB‐mediated cytoplasmic polyadenylation in neurons (Du & Richter, 2005) and identify transcripts that were polyadenylated by the cytoplasmic poly(A) polymerase GLD4 (Shin et al., 2017). Using this method, a positive correlation was found between poly(A) tail length and Pab1 and ribosome density in yeast (Beilharz & Preiss, 2007). To study differential polyadenylation at different cell cycle stages in HeLa cells (Novoa et al., 2010), a mixed approach was applied. Poly(U) chromatography with elution at low temperature selects RNAs with short poly(A) tails, whereas the oligo(dT) approach isolates all polyadenylated RNAs. These two fractions were compared (Novoa et al., 2010).

These methods are able to detect changes in poly(A) tail lengths in a transcriptome‐wide manner, but they do not provide information about the exact poly(A) tail length. Thus, subtle changes in poly(A) tail length can be missed, and information about very short poly(A) tails is lacking. Moreover, the utilization of microarrays has several drawbacks, especially compared with RNA sequencing (RNA‐Seq). Only genes to which probes are designed can be detected, and nonspecific hybridization and cross‐hybridization impede the results (S. Zhao et al., 2014). Overall, although they made a step forward compared with previous single‐transcript approaches, leading to many biologically relevant observations, they still do not meet the rising demands of the research community.

## 4 POLY(A) TAIL LENGTH MEASUREMENT BY SEQUENCING

The development of NGS with the RNA‐Seq technique raised new possibilities in transcriptomic studies. Starting from simple differential gene expression analysis at a transcriptome‐wide scale, the applications of high‐throughput sequencing are still rising, including polyadenylation status analyses. Here, we review methods of poly(A) tail length measurements using Illumina, PacBio, and ONT sequencing platforms.

### 4.1 Illumina‐based approaches: TAIL‐Seq, PAL‐Seq, and m‐TAIL‐Seq

Since the development of RNA‐Seq in 2008, the Illumina platform has been a leading technology in the field. The basis of Illumina RNA‐Seq is the generation of libraries of short cDNA fragments with adapters on one or both ends from mRNA‐enriched or rRNA‐depleted RNA, which are then amplified by PCR. cDNA molecules are clustered on a flow cell and sequenced from one end (single‐end sequencing) or both ends (paired‐end sequencing) by reading the fluorescence signal from a 3′‐blocked fluorescently labeled nucleotide that is incorporated during DNA synthesis (so‐called “sequencing‐by‐synthesis”; Stark et al., 2019). This approach is most commonly used for differential expression analysis, but library preparation and data analysis workflows are highly adjustable and compatible with various applications, such as protocols for poly(A) tail sequencing.

**FIGURE 3:**
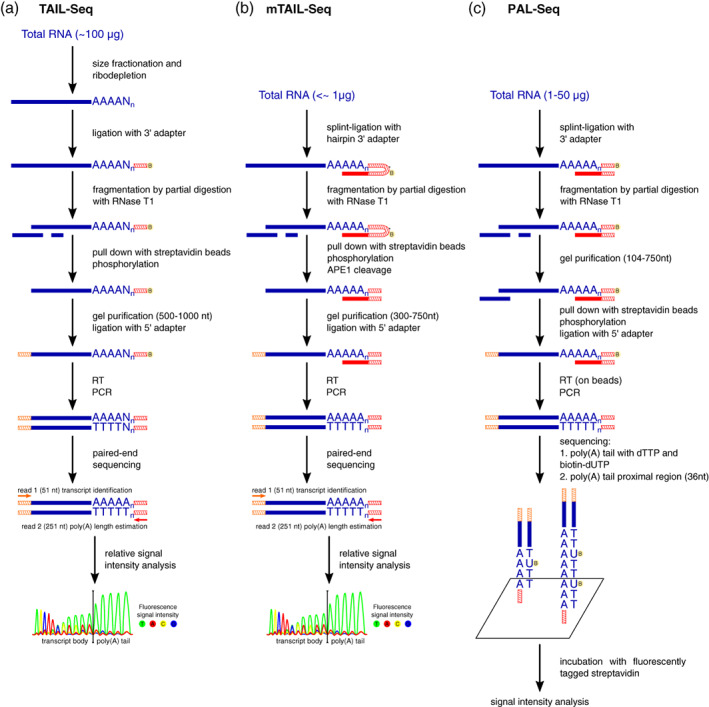
Comparison of TAIL‐Seq, mTAIL‐Seq, and PAL‐Seq protocols for mRNA poly(A) tail sequencing. The red line indicates the oligo(dT) sequence. Red hatched lines indicate the 3′ adapter sequence. Orange hatched lines indicate the 5′ adapter sequence. The “B” in the yellow circle indicates biotin. Asterisks indicate an abasic site in the 3′ adapter.

The first approaches to measure poly(A) tail lengths at genomic scale were published in 2014, including poly(A)‐tail length profiling by sequencing (PAL‐Seq; Subtelny et al., 2014) and TAIL‐Seq (Chang et al., 2014). The latter was subsequently modified to become mRNA TAIL‐Seq (mTAIL‐Seq; Lim et al., 2016). Both PAL‐Seq and TAIL‐Seq rely on the preparation of sequencing libraries with intact mRNA 3′ ends, including the poly(A) tail, which is further sequenced on the Illumina platform. Although these approaches differ in their sequencing procedures and computational approaches that are used for the determination of poly(A) tail length, they utilize very similar experimental procedures (see overview in Figure 3). RNA is first ligated with a biotinylated 3′ DNA adapter and partially digested with RNase T1 that cleaves RNA after guanine residues, thereby leaving intact poly(A) tails. Subsequent pull‐down on streptavidin beads allows the enrichment of only 3′ end fragments. Next, size selection by gel electrophoresis and ligation with a 5′ adapter provide complete templates for reverse transcription, library amplification, and sequencing on the Illumina platform (Chang et al., 2014; Lim et al., 2016; Subtelny et al., 2014). The order of steps slightly differs among these procedures, but one of the main differences between them is the form of the 3′ adapter. In TAIL‐Seq, it is a single‐stranded DNA adapter, thus introducing the rRNA‐depletion step as necessary for the analysis of only poly(A) + molecules (Chang et al., 2014). In PAL‐Seq, the adapter is also a single‐stranded DNA, but a second DNA oligonucleotide that is complementary to both the poly(A) tail and 3′ adapter fragment facilitates efficient ligation (i.e., splint ligation) to exclusively mRNA molecules, thus eliminating the need for ribodepletion (Subtelny et al., 2014). In mTAIL‐Seq, the adapter and splinter create one hairpin oligonucleotide that improves ligation efficiency. Additionally, two abasic sites that are introduced in this hairpin enable an efficient cut from streptavidin beads by apurinic/apyrimidinic endonuclease 1 (APE1; Lim et al., 2016).

A major difference between PAL‐Seq and TAIL‐Seq lies in the sequencing procedure and bioinformatic analysis. PAL‐Seq is compatible only with the discontinued Illumina Genome Analyzer sequencer, whereas TAIL‐Seq was successfully applied on Hiseq2000 and MiSeq machines. In the case of PAL‐Seq on a flow cell, after cluster generation, a sequencing primer that is complementary to the 3′ adapter is extended with a mixture of dTTP and biotinylated dUTP to label the poly(A) tail, and standard Illumina chemistry is subsequently applied to sequence the fragment upstream of poly(A), thereby allowing transcript identification. Because of incubation with fluorochrome‐conjugated streptavidin, the poly(A) tail length is estimated from the fluorescence signal intensity that is normalized to cluster density, assuming that the amount of incorporated biotin‐dUTP increases proportionately to poly(A) tail length. In the original protocols for TAIL‐Seq and mTAIL‐Seq, paired‐end sequencing allowed identification of the transcript from read 1 (51 nt) and examination of the poly(A) tail with read 2 (231 nt), but these read lengths can be customized. Standard Illumina sequencing fails when analyzing long homopolymer tracts. Therefore, the authors of TAIL‐Seq developed an algorithm that measures the length of the poly(A) tail using fluorescence quantification. Importantly, this requires modification in the sequencer's control software, which allows storing intermediate CIF files that contain cluster fluorescence intensity information. When analyzing read 2, the polyadenylation site is detected by analyzing changes in relative fluorescence, defined as the ratio of the signal from thymidine (i.e., in the second read, the complementary cDNA strand is sequenced, thus obtaining the poly[T] homopolymer) to the signal from the different base. After the long poly(T) stretch, a signal from thymidine is still detectable even when a non‐T base is sequenced, but the relative fluorescence changes, leading to the identification of the poly(A) tail start site and allowing calculation of the poly(A) tail length (Chang et al., 2014; Lim et al., 2016). In both approaches, accurate measurements are based on comparisons to signals from spike‐ins with defined poly(A) tail lengths (Subtelny et al., 2014).

Although these methods provide nucleotide‐resolution poly(A) tail length measurements on a genomic scale, they clearly have limitations (Table 1). Both reverse transcription and PCR amplification steps can introduce bias, leading to underestimations of the length of poly(A) tails. Because poly(A) spike‐ins are generated by PCR or in vitro polyadenylation, they provide a range of poly(A) tail lengths rather than exact lengths. TAIL‐Seq initially requires a large amount of RNA (~100 μg; Lim et al., 2016) and expensive rRNA depletion (Chang et al., 2014). In mTAIL‐Seq and PAL‐Seq, the splint ligation step reduces the amount of starting material to as low as 100 ng (Lim et al., 2016) or 1–50 μg (Subtelny et al., 2014) of RNA and captures specifically poly(A) + mRNAs without a need for ribodepletion. However, a disadvantage of splint ligation is the absence of data on 3′ end modifications, such as uridylation and guanylation, which can still be identified with TAIL‐Seq (Chang et al., 2014). Because very short and modified poly(A) tails are not captured, longer median poly(A) lengths are also obtained (Lim et al., 2016). As a result, there are discrepancies in median poly(A) tail lengths between TAIL‐Seq and PAL‐Seq (Table 2). Although all of these methods utilize the Illumina sequencing system, PAL‐Seq requires manipulations on a flow cell, thereby introducing an additional imaging step, and also requires the now‐discontinued Genome analyzer II sequencer, which in reality makes this method impractical. Both TAIL‐Seq and mTAIL‐Seq can be performed on the Illumina HiSeq or MiSeq platform. Because of the higher sensitivity of mTAIL‐Seq, a lower sequencing depth from small‐scale experiments on MiSeq can provide sufficient data while reducing the overall cost of the experiment (Lim et al., 2016). Importantly, both methods present significant bioinformatic challenges and are difficult to implement. For PAL‐seq, raw signal intensities from the flow cell are collected and further processed to calculate poly(A) length for each cluster on the flow cell. In the case of TAIL‐seq and its derivatives, raw intensities are analyzed by the provided software (tailseeker; https://github.com/hyeshik/tailseeker), which has multiple dependencies and is no longer maintained. There is also a known issue in the latest version of tailseeker that is related to the patterned imbalance of fluorescence signals among the channels and problems with signal normalization. To the best of our knowledge, these issues remain unresolved, as this software is not currently being developed.

**TABLE 1:** Summary of major differences between the most common 3′‐end sequencing approaches

Feature	Method					
	*TAIL‐Seq* (Chang et al., 2014)	*mTAIL‐Seq* (Lim et al., 2016)	*PAL‐Seq* (Subtelny et al., 2014)	*FLAM‐Seq* (Legnini et al., 2019)	*PAIso‐Seq* (Y. Liu, Nie, et al., 2019)	*DRS* (Garalde et al., 2018)
RNA preparation	Ribodepletion	Not required	Not required	Poly(A) + selection	Not required	Poly(A) + selection
Amount of starting material	100 μg	<1 μg	1–50 μg	500 ng–10 μg	≤100 ng	500 ng of polyadenylated RNA
Sequencing platform	Illumina HiSeq/MiSeq	Illumina HiSeq/MiSeq	Illumina Genome Analyzer	Pacific BioSciences	Pacific BioSciences	Oxford Nanopore Technologies
3′ adapter addition	Ligation of biotinylated single‐stranded DNA oligo	Splint ligation of biotinylated hairpin DNA oligo	Splint ligation of biotinylated single‐stranded DNA oligo in the presence of splint DNA oligo	Enzymatically added G/I tail serving as anchor for oligo	3′‐end extension of template oligo containing T stretch	Splint ligation of DNA oligo containing T stretch
Fragmentation	Partial digestion with RNase T1	Partial digestion with RNase T1	Partial digestion with RNase T1	None	None	None
PCR bias	+	+	+	+	+	−
RT bias	+	+	+	+	+	−
Oligo(dT) selection bias	−	−	−	+	−	+
Error rate	Low	Low	Low	Low	Low	High
Read length	Short	Short	Short	Long	Long	Long
Detection of modifications in 3′‐end of poly(A) tail	+	−	−	−	−	−
Detection of modifications within poly(A) tail	−	−	−	+	+	−

**TABLE 2:** Comparison of results obtained with mRNA 3′‐end sequencing methods, including median poly(A) tail length and correlations between poly(A) tail length and mRNA expression, half‐life, and translational efficiency

Model	Method	Median poly(A)		Correlation with expression	Correlation with half‐life	Correlation with translation efficiency
		Total [nt]	Intragenic [nt]			
HeLa	TAIL‐Seq (Chang et al., 2014)	59	60			*R*_*p*_ = 0.058noncano
	PAL‐Seq (Subtelny et al., 2014)	67.5	82.6	*R*_*s*_ = −0.053	*R*_*s*_ = −0.048	*R*_*s*_ = −0.1
	Poly(A)‐Seq (Yu et al., 2020)	95.5				
HeLa S3	FLAM‐Seq (Legnini et al., 2019)	81	87	*R*_*p*_ = −0.18	*R*_*p*_ = −0.13	*R*_*p*_ = −0.1
HEK923T	PAL‐Seq (Subtelny et al., 2014)	75.3	88.4	*R*_*s*_ = −0.23		*R*_*s*_ = 0.07
HCT116	Poly(A)‐Seq (Yu et al., 2020)	101.5				
K562	DRS (Maier et al., 2020)	94.75			*R*_*s*_ = 0.48	
Human iPSC	FLAM‐Seq (Legnini et al., 2019)	97	111.5	*R*_*p*_ = −0.19		
Cerebral organoids	FLAM‐Seq (Legnini et al., 2019)	104	121.5	*R*_*p*_ = −0.32		
NIH3T3	TAIL‐Seq (Chang et al., 2014)	60	61	*R*_*p*_ = 0.076	*R*_*p*_ = 0.364	*R*_*p*_ = 0.009
	PAL‐Seq (Subtelny et al., 2014)	95.7	107.8	*R*_*s*_ = −0.23	*R*_*s*_ = −0.16	*R*_*s*_ = −0.04
Mouse liver	PAL‐Seq (Subtelny et al., 2014)	66.6	75.3	*R*_*s*_ = 0.085		*R*_*s*_ = 0
Mouse	Nano3P‐Seq (Begik et al., 2021)	100.7	106.4			
Rat liver	PAIso‐Seq (Y. Liu, Nie, et al., 2019)	63				
*Arabidopsis* leaf	PAL‐Seq (Subtelny et al., 2014)	50.7	58.1			
*Arabidopsis*	DRS (Parker et al., 2020)	68		*R*_*s*_ = −0.34		
*Drosophila* S2	PAL‐Seq (Subtelny et al., 2014)	50.4	63.3			
*Drosophila* Immature oocyte Mature oocyte Activated egg	mTAIL‐Seq (Lim et al., 2016)	60 75 73	58 76 70			NA *R*_*s*_ = 0.306 *R*_*s*_ = 0.638
*S*. *cerevisiae*	PAL‐Seq (Subtelny et al., 2014)	26.7	33.1	*R*_*s*_ = −0.44	*R*_*s*_ = 0.048 *R*_*s*_ = −0.094 *R*_*s*_ = 0.044 *R*_*s*_ = −0.11 *R*_*s*_ = 0.045 *R*_*s*_ = −0.44 *R*_*s*_ = 0.23 *R*_*s*_ = −0.35	*R*_*s*_ = −0.12
	DRS (Tudek et al., 2021)	40		*R*_*p*_ = −0.53	*R*_*p*_ = −0,22 *R*_*p*_ = −0.39	
	Nano3P‐Seq (Begik et al., 2021)	22.3	24.8			
*S*. *pombe*	PAL‐Seq (Subtelny et al., 2014)	28.2	32.8	*R*_*s*_ = −0.31		*R*_*s*_ = −0.15
Mouse germinal vesicle stage oocytes	PAIso‐Seq (Y. Liu, Nie, et al., 2019)	55.3	55.7			
*Danio rerio*	Nano3P‐Seq (Begik et al., 2021)	74.5	63.6			
*Danio rerio* embryo: 2 hpf 4 hpf 6 hpf	PAL‐Seq (Subtelny et al., 2014)	23.1 42.2 58.1	29.4 44 58.9	*R*_*s*_ = −0.13 *R*_*s*_ = −0.011 *R*_*s*_ = −0.25		*R*_*s*_ = 0.77 *R*_*s*_ = 0.62 *R*_*s*_ = 0.13
*X*. *laevis* embryo: Stage 3–4 Stage 9 Stage 12–12.5	PAL‐Seq (Subtelny et al., 2014)	21.7 35.4 44.5	25.4 38.1 49.2			*R*_*s*_ = 0.63 *R*_*s*_ = 0.65 *R*_*s*_ = 0.13
*C*. *elegans* L4 larval stage	mTAIL‐Seq (Lima et al., 2017)	57	82	*R*_*s*_ = −0.24		*R* = −0.32 F_op_ instead of TE
	FLAM‐Seq (Legnini et al., 2019)	51	59	*R*_*p*_ = −0.19		
*C*. *elegans* adult stage	FLAM‐Seq (Legnini et al., 2019)	44.5	49.5	*R*_*p*_ = −0.21		

Abbreviations: DRS, Nanopore direct RNA sequencing; *F*_op_, frequency of optimal codons; *R*_p_, Pearson's correlation coefficient; *R*_s_, Spearman's correlation coefficient; TE, translational efficiency.

*Note*: In the case of poly(A) tail length, in poly(A)‐Seq, FLAM‐Seq, and PAIso‐Seq, the mean of medians for replicates is given.

The utilization of TAIL‐Seq and PAL‐Seq has dramatically expanded our knowledge of 3′ ends of mRNA. Poly(A) tails are shorter than previously considered (>200 nt; Weill et al., 2012), with a median poly(A) tail length of ~60 nt (Chang et al., 2014) or in the range of 67–96 nt (Subtelny et al., 2014) in various mammalian cells (summarized in Table 2). Their lengths in orthologous genes are conserved among species (Subtelny et al., 2014). Indeed, shorter poly(A) tails occur in highly expressed genes (Lima et al., 2017; Subtelny et al., 2014), and the length correlates positively with translation efficiency in maturating oocytes and early embryonic development until gastrulation (Eichhorn et al., 2016; Lim et al., 2016; Subtelny et al., 2014; Yang et al., 2020) while in somatic cells only when the poly(A) tail is shorter than 20 nt (Park et al., 2016). In other cases, such a correlation does not exist (Chang et al., 2014; Park et al., 2016; Subtelny et al., 2014), or it is even negative with the frequency of optimal codons in *C*. *elegans* (Lima et al., 2017; Table 2). Interestingly, a polyadenylation shift occurs during oocyte maturation in *Drosophila* (Lim et al., 2016) and *Xenopus laevis* (Yang et al., 2020) and during embryo development in *Danio rerio* and *X*. *laevis* (Subtelny et al., 2014). In addition to polyadenylation, 3′ ends of RNA are frequently uridylated with a preference for shorter poly(A) tails or less commonly guanylated with a preference for longer poly(A) tails, which correlates negatively and positively, respectively, with half‐life (Chang et al., 2014; Eisen, Eichhorn, Subtelny, Lin, et al., 2020; Lim et al., 2018). Indeed, TAIL‐Seq analysis revealed that TUT4 and TUT7 are enzymes that catalyze the uridylation of mRNAs with poly(A) tails <25 nt, which facilitates mRNA decay (Lim et al., 2014). This constitutes an important degradation mechanism of maternal transcripts in growing oocytes (Morgan et al., 2017) and early developmental stages in vertebrates but not invertebrates (Chang et al., 2018). Widespread uridylation was also detected in plants (Zuber et al., 2016). With the mTAIL‐Seq approach, the deadenylases poly(A)‐specific ribonuclease (PARN) and target of EGR1 (TOE1) do not act on the poly(A) tail of mRNA, contributing to nuclear small‐noncoding RNA maturation (Son et al., 2018).

Recently, the novel PAL‐Seq version 2 (v2) approach that combines the advantages of PAL‐Seq and TAIL‐Seq was published (Eisen, Eichhorn, Subtelny, Lin, et al., 2020). This approach utilizes splint ligation like PAL‐Seq but is modified to enable the capture of poly(A) tails with terminal U. Poly(A) tails are measured by direct sequencing as in TAIL‐Seq, without additional labeling steps, like in the original PAL‐Seq approach. Application of this approach, combined with mRNA labeling with 5‐ethynyl uridine in the time course, provided insights into poly(A) tail length dynamics in the cytoplasm and revealed their shortening toward a steady‐state length over time and a positive correlation between poly(A) tail length and mRNA half‐life after 2 h of labeling (pre‐steady‐state; Eisen, Eichhorn, Subtelny, Lin, et al., 2020), which was previously undetectable when steady‐state levels of mRNA were analyzed (Chang et al., 2014; Eisen, Eichhorn, Subtelny, Lin, et al., 2020; Subtelny et al., 2014). PAL‐Seq v2 was also used to study the dynamics of miRNA‐mediated deadenylation, revealing its effect only on young mRNAs, whereas steady‐state poly(A) tail lengths remained unaffected (Eisen, Eichhorn, Subtelny, & Bartel, 2020a). This method is still being improved in slightly differing variants (PAL‐Seq v3 and v4; Xiang & Bartel, 2021). It was also combined with translating ribosome affinity purification (TRAP) to create poly(A) tail‐length profiling following TRAP (PAL‐TRAP) to study intragenic effects of poly(A) tail length on translational efficiency (Xiang & Bartel, 2021). A combination of these protocols was used to elucidate the role of PABPC1 in coupling mRNA poly(A) tail length with translational efficiency in frog oocytes (Xiang & Bartel, 2021).

The publication of these methods broadened our ability to study poly(A) tails. Data that have been generated with these methods changed our view of poly(A) tail length and its relationship to different aspects of RNA metabolism. However, in addition to the aforementioned disadvantages of PCR‐biased estimations of tail length, these protocols are technically challenging and not commonly used among RNA‐focused labs. The PAL‐Seq protocol requires discontinued equipment, and further improvements to this protocol make it similar to TAIL‐Seq. From our experience, library preparation for TAIL‐Seq is not trivial and requires several steps of optimizations. It is also challenging to obtain high reproducibility between repetitions (Warkocki et al., 2018). Other problems include the requirement for a large amount of starting material and a costly ribodepletion step. Combined, this makes the protocol difficult, time‐consuming, and expensive. Thus, in addition to the undeniable importance of these protocols, further developments in the field of poly(A) tail sequencing were essential for further progress in the field.

### 4.2 Other Illumina‐based methods

**FIGURE 4:**
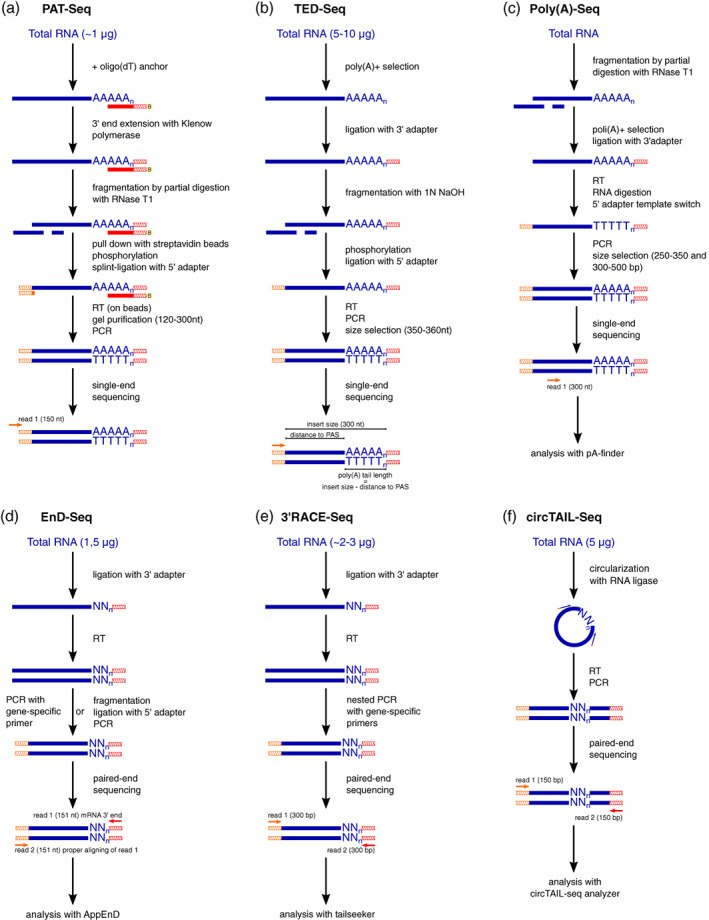
Overview of other Illumina‐based approaches to sequence RNA 3′ ends. The red line indicates the oligo(dT) sequence. Red hatched lines indicate the 3′ adapter sequence. Orange hatched lines indicate the 5′ adapter sequence. The “B” in the yellow circle indicates biotin attached to the 3′ adapter.

After these first achievements in genome‐wide poly(A) tail length measurements, many other, albeit less common, methods to measure poly(A) tails at genomic scale based on sequencing with the Illumina platform were published. One of them is poly(A)‐test RNA sequencing (PAT‐Seq; Harrison et al., 2015). Figure 4a shows a general overview of PAT‐Seq, which is similar to the previously described TAIL‐Seq and PAL‐Seq. It utilizes the Klenow fragment of DNA polymerase I to extend the 3′ end of RNA with dNTPs on a template of annealed biotinylated anchored adapter oligonucleotide instead of adapter ligation. It also employs standard Illumina sequencing procedures and directional sequencing from the 5′ end as a solution to a problem of fluorescence signal blurring after a long homopolymer stretch. The poly(A) site is detected as the first non‐templated adenosine in a tract that is longer than 3 A (Harrison et al., 2015).

The correlation between poly(A) tail data from PAT‐Seq and PAL‐Seq (Subtelny et al., 2014) is only 0.3339, with only a 1 nt difference in average poly(A) length. However, employed in this study, the sequencing of only 100 bases prevents the detection of poly(A) tails that are longer than 80 nt (Harrison et al., 2015). Even in a yeast model, where poly(A) tails are rather short (Subtelny et al., 2014), this can lead to the loss of information about the maximal poly(A) tail and restrict its applications. Improvements of this approach by utilizing 150 bp sequencing (Swaminathan et al., 2019) occurred in studies of *C*. *elegans* (Elewa et al., 2015). This method was also used as standard RNA‐Seq for differential expression analysis (Beilharz et al., 2017; Harrison et al., 2015; Johnstone et al., 2018; Tucey et al., 2018), showing that data from PAT‐Seq and RNA‐Seq correlated well (*r* = 0.7860; Harrison et al., 2015).

Another method for transcriptome‐wide poly(A) tail length profiling is tail‐end displaced sequencing (TED‐Seq; Woo et al., 2018). Similar to TAIL‐Seq, poly(A) + RNA is subjected to ligation with a 3′ adapter, fragmentation, and ligation with a 5′ adapter, followed by RT‐PCR. The most important step (i.e., size selection by gel excision) must be precisely performed, and libraries are sequenced using the standard Illumina platform (Figure 4b). The 5′ ends are mapped to a reference genome, and 3′ cleavage and polyadenylation sites are determined. The distance between the 5′ end and 3′ polyadenylation site is subtracted from the total size of the fragments, thus giving the poly(A) tail length. Thus, the analysis does not require sophisticated algorithms or modifications of the sequencing run, making it cost‐efficient. In this approach, a 100‐bp read was used to determine 3′ cleavage and the polyadenylation site, and a 300‐bp read was used for poly(A) tail length estimations. To determine absolute poly(A) tail length, in vitro synthesized spike‐ins of known poly(A) tail length were used for normalization (Woo et al., 2018). The major limitation of this protocol, however, is the size selection step, which must be done precisely and can directly affect the results. Although the resolution was determined to be ~10 bp (Woo et al., 2018), it may vary between laboratories. Because the recovery rate can differ between samples (Woo et al., 2018), this method cannot be used for quantitative analysis. Additionally, the poly(A) + selection step using oligo(dT) beads can introduce bias into RNAs with longer poly(A) tails (Park et al., 2016).

Poly(A) tail lengths in HEK293 cells, measured by TED‐Seq, modestly correlate with data from PAL‐Seq (*R* = 0.27; *R* = 0.22 for correlation between PAL‐Seq and TAIL‐Seq; Woo et al., 2018). This method was used for the analysis of poly(A) tails in the endoplasmic reticulum stress response, showing that under this specific condition, poly(A) tails of stress‐responsive transcripts are lengthened, which positively correlates with the stability and translational de‐repression of these mRNAs (Woo et al., 2018).

The most recent method of poly(A) tail sequencing with Illumina is poly(A)‐Seq (Yu et al., 2020). This protocol starts with RNA fragmentation by partial digestion with RNAse T1. RNA fragments are subjected to the oligo(dT) selection of polyadenylated fragments, which are subsequently ligated to a single‐stranded 3′ adapter. It is unlike TAIL‐Seq and PAL‐Seq protocols that start with the adapter ligation step. Authors that used poly(A)‐Seq claim that ligation after fragmentation is more efficient. RNA is then reverse‐transcribed, followed by second‐strand synthesis based on template‐switching for 5′ adapter ligation. cDNA is amplified and sequenced (Figure 4c) with a NextSeq 500 to obtain 300‐nt‐long single reads. Authors also developed pA‐finder, a pipeline for poly(A) tail length analysis. It starts by searching for stretches of adenosines (9As and 6As closest to the 5′ and 3′ ends, respectively), and the region between them is considered to be a poly(A) tail. Only reads that contain such a fragment are subjected to further analysis. The sequence next to the 5′ end is mapped to the genome, and the poly(A) end position is determined by quality assessment.

Poly(A) tails that are measured using this method are longer than tails that are measured with TAIL‐Seq and PAL‐Seq (median poly[A] tail length for HeLa cells: 93–98 nt; median poly[A] tail length for HCT116 cells: 101–102 nt; median poly[A] tail length for HEK293 cells: 100–103 nt) (Yu et al., 2020). Nonetheless, this method, similar to all methods that utilize short reads) depletes the population of very long poly(A) tails (>150 nt). Correlations between library size and median poly(A) tail length were analyzed. Libraries of large fragments (350–450 nt) and small fragments (250–350 nt) yielded a significant difference in poly(A) tail lengths (~30–120 nt for large fragments vs. ~30–80 nt for small fragments), indicating that the size selection step has a profound impact on the outcome and can explain different lengths that are obtained with different methods. However, because poly(A)‐Seq relies on oligo(dT) selection, it is biased toward longer poly(A) tails. Furthermore, in the pA‐finder pipeline, preliminary poly(A) tails that are shorter than 10 nt are discarded, thus losing information about very short poly(A) tails (Yu et al., 2020). Poly(A)‐Seq enables the detection of other nucleotides in poly(A) tails and has been used to detect guanosines in poly(A) tails of *Arabidopsis thaliana* samples (T. Zhao, Huan, et al., 2019).

All of the above described methods focus on the measurement of long poly(A) tails, and they either do not capture (Lim et al., 2016) or exclude from the analysis (Chang et al., 2014) poly(A) tails that are very short and non‐poly(A) tails. Exonuclease degradation sequencing (EnD‐Seq; Welch et al., 2015) is a sequencing protocol that detects 3′‐end modifications of non‐polyadenylated RNAs or decay intermediates, even 1 nt long. To preserve 3′ ends, total RNA is ligated with the 3′ adapter, followed by cDNA synthesis from the primer antisense to the adapter. To increase sensitivity, this adapter can have a 3′ end that is designed to detect certain 3′ ends of interest. After fragmentation and 5′ adapter ligation, the library is amplified and sequenced (Figure 4d). Because of the utilization of Illumina paired‐end sequencing, read 1 contains the adapter sequence and RNA 3′ while read 2 helps with proper alignment. To analyze the data, the application for mapping EnD‐Seq data (AppEnD; Welch et al., 2015) computational method was developed. Unlike most approaches, AppEnD does not utilize the prealignment stripping of homopolymers from raw reads, identifying nontemplated 3′ end modifications as parts of reads that do not match the reference genome or the linker during alignment (i.e., soft‐clipped reads).

Despite its application for short poly(A) tail detection, this method cannot be used for long poly(A) tail analysis. Importantly, improvements in sensitivity with specifically designed primers affect the results, in which the utilization of primers that end with 3 A cannot detect U‐tails that are shorter than 3 nt (Welch et al., 2015).

EnD‐Seq combined with the AppEnD approach was used mainly to study the role of uridylation in non‐polyadenylated histone mRNA metabolism (Holmquist & Marzluff, 2019; Lackey et al., 2016; Meaux et al., 2018; Slevin et al., 2014; Welch et al., 2015). The AppEnD algorithm that was independently used in the EnD‐Seq protocol was used to precisely measure poly(A) tail lengths of tails up to 15 nt based on data on *Drosophila* short capped RNAs (Henriques et al., 2013) and up to 30 nt from TAIL‐Seq (Chang et al., 2014) raw data (Welch et al., 2015). Alternatively, EnD‐Seq can be used for the deep analysis of specific transcripts (or groups of transcripts) if PCR with a gene‐specific primer that contains a 5′ adapter sequence replaces standard adapter ligation (Welch et al., 2015).

Similar to EnD‐Seq, 3′RACE‐Seq (Goldfarb & Cech, 2013; Warkocki et al., 2018; Wilusz et al., 2011) allows the analysis of short 3′ termini of ncRNAs but only in a transcript‐specific manner. This method utilizes a modified 3′RACE protocol to perform the deep sequencing of RNA 3′ ends. RNA is ligated to a 3′ adapter. After reverse transcription from an adapter‐specific primer, libraries for Illumina sequencing are generated by nested PCR (Figure 4e). The detection of 3′‐end modifications is based on filtering the reads by aligning both to the 3′ region of a gene and adapter sequence (Goldfarb & Cech, 2013). The 3′RACE‐Seq approach was used in studies of RNase MRP 3′ (Goldfarb & Cech, 2013), tRNA and tRNA‐like small RNAs (Wilusz et al., 2011), and human telomerase RNA (L. Chen et al., 2020; Gable et al., 2019) 3′ termini or combined with a bioinformatical pipeline based on TAIL‐Seq (Chang et al., 2014) to study the role of uridylation by TUT4/TUT7 on LINE‐1 retrotransposition (Warkocki et al., 2018).

A variant of 3′RACE‐Seq was recently published: Nascent RNAend‐Seq (Roake et al., 2019). This method combines nascent RNA‐Seq (Rädle et al., 2013) and 3′ RACE‐Seq (Goldfarb & Cech, 2013) to study changes in ncRNA 3′ tails during RNA maturation. Cells are treated with the nucleotide analog 4‐thiouridine (4SU), which labels newly transcribed RNA. After thiol‐specific biotinylation, nascent RNA is purified on streptavidin beads. RNA is then subjected to 3′ adapter ligation, followed by reverse transcription from a primer that contains an adapter sequence and PCR with a gene‐specific primer and universal primer that contains the adapter sequence. Libraries are sequenced using the standard Illumina platform. This method has a strikingly different application from the ones described above, but it was successfully used to define the role of oligoadenylation by PAP‐associated domain‐containing protein 5 (PAPD5) and deadenylation by poly(A)‐specific ribonuclease (PARN) in human telomerase RNA maturation (Roake et al., 2019).

Another approach to measure poly(A) tail length by targeted sequencing of the only transcript of interest was described as circTAIL‐Seq (Gazestani et al., 2016). This method combines low‐throughput circRT‐PCR (Couttet et al., 1997) with NGS. Total RNA is circularized by RNA ligase, followed by RT‐PCR with primers that are designed to obtain a 3′–5′ junction of a transcript of interest (Figure 4f). Because primers contain an adapter sequence, after these steps, a template for sequencing is generated. This method utilizes standard Illumina paired‐end sequencing, and data are analyzed using an informatics workflow to allow estimations of poly(A) tail length and composition (Gazestani et al., 2016). circTAIL‐Seq is used for single‐transcript analysis and low‐abundance mRNAs. Importantly, circTAIL‐Seq was employed for mitochondrial transcript analyses from *Trypanosoma brucei*, where short (<30 nt) and heterogeneous poly(A) tails are typical (Gazestani et al., 2016; Gazestani et al., 2018). Therefore, circTAIL‐Seq applications for longer poly(A) tails that consist of only adenosines is currently untested, especially because no poly(A) tail length spike‐ins were used in the original study (Gazestani et al., 2016).

In 2018, a completely new approach to generate mRNA 3′ ends for sequencing that is based on tagmentation by Tn5 transposase was published (Hennig et al., 2018). This protocol utilizes a previously published Tn5 tagmentation approach (Adey et al., 2010; Picelli, Björklund, et al., 2014) that is used in the Nextera XT library preparation kit from Illumina. A hyperactive mutated version of Tn5 that is loaded with two different linker sequences cuts double‐stranded DNA, thus ligating linker sequences to both ends of the cut. Linkers are composed of a mosaic end (Zhou et al., 1998) that is recognized by Tn5 and overhangs that are templates for index adapter primers for PCR. Tagmentation generates short DNA fragments that are surrounded by known sequences that are used as templates for primers during amplification (Adey et al., 2010). cDNA is synthesized with an oligo(dT) primer that contains a 3′ adapter sequence, and the second strand is generated by a previously published template‐switching protocol (Picelli, Faridani, et al., 2014). Tn5 that is loaded with only one linker sequence performs double‐stranded cDNA tagmentation, leading to (*i*) mRNA 3′ end fragments that are surrounded by a 5′ adapter in the Tn5 linker and 3′ adapter in the oligo(dT) primer and (*ii*) fragments from the mRNA body that are surrounded only by linkers. Thus, PCR with primers for 5′ and 3′ adapters that omits the gap‐filling step allows the specific amplification of only 3′‐end sequences (Hennig et al., 2018).

This protocol is fast, robust, and inexpensive and requires only a low amount of starting material. Its main disadvantage is a lack of strand specificity. To date, this method has been used only for poly(A) site mapping. Nevertheless, this mRNA 3′ end library preparation protocol and its derivatives (e.g., transposase‐mediated 3′‐RNA‐Seq [TM3′‐Seq]; Pallares et al., 2020), combined with a good approach for sequencing and data analysis, can be useful for polyadenylation studies. However, no such protocol has yet been published. Thus, this approach requires a significant optimization effort. We tried performing 3′ end and standard RNA‐Seq with Tn5. We found that although standard RNA‐Seq works well, effective tagmentation and amplification of the mRNA 3′ end is challenging.

In summary, over the last 8 years, much progress has been made in the area of poly(A) tail length measurement techniques by designing protocols that are based on NGS. Various protocols have been created, most notably TAIL‐Seq and PAL‐Seq. Each of these techniques has its own advantages and disadvantages, with the common drawback of poly(A) tail estimation that is biased by the PCR amplification of long homopolymer stretches. However, these methods were designed based on the needs of specific laboratories, and some have never been used beyond the laboratory that developed them. Moreover, some of the methods were designed to analyze single transcripts or short poly(A) tails, thus limiting their application. Furthermore, the data that were generated with these protocols for sequencing, including TAIL‐Seq and PAL‐Seq, do not correlate well, raising the important problem of a lack of reproducibility between laboratories and questions about the reliability of estimations of these methods. When considering the technical challenges, cost, and time needed for library preparation, only a few laboratories have successfully performed poly(A) tail sequencing. Thus, there is a need for an easy‐to‐implement protocol that generates reproducible results.

### 4.3 Poly(A) tail length measurement with the PacBio platform

Although Illumina‐based approaches dominate in transcriptomic studies, two research groups published the first successful poly(A) tail profiling protocols using the PacBio third‐generation sequencing platform: full‐length mRNA sequencing (FLAM‐Seq; Legnini et al., 2019) and poly(A) inclusive RNA isoform sequencing (PAIso‐Seq; Y. Liu, Nie, et al., 2019). Unlike the Illumina sequencing‐by‐synthesis of clustered short cDNA fragments approach, PacBio features single‐molecule, real‐time (SMRT) sequencing (Eid et al., 2009) to generate full‐length cDNA sequences with Iso‐Seq technology (Gonzalez‐Garay, 2016). The utilization of circular consensus sequencing by the multiple passing of a single template molecule generates long high‐fidelity reads with high accuracy (even 99.8%; Wenger et al., 2019).

**FIGURE 5:**
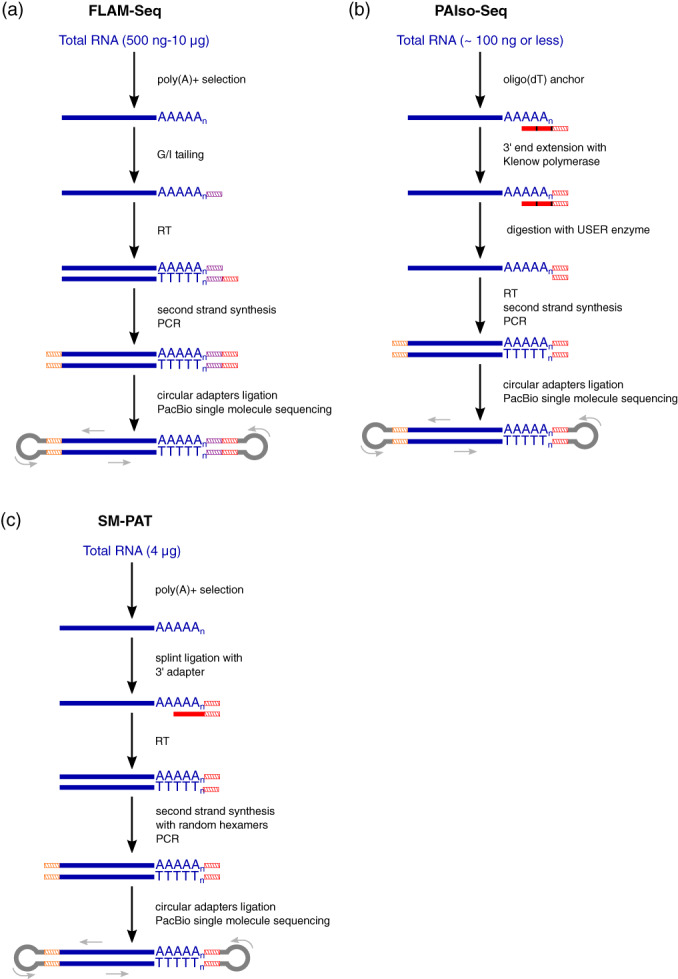
Comparison of FLAM‐Seq, PAIso‐Seq, and SM‐PAT protocols for mRNA poly(A) tail sequencing with the PacBio platform. Violet hatched lines indicate the G/I tag sequence. Red hatched lines indicate the 3′ adapter sequence. Orange hatched lines indicate the 5′ adapter sequence. The red line indicates the oligo(dT) sequence. dU nucleotides are marked in black.

The FLAM‐Seq (Legnini et al., 2019) and PAIso‐Seq (Y. Liu, Nie, et al., 2019) protocols present very similar approaches. The PacBio platform, which can handle homopolymers better than Illumina, enables sequencing of the whole molecule of polyadenylated RNA, providing isoform‐specific information about poly(A) tails. The main steps of both protocols are 3′‐end extension, RT with template switching, cDNA amplification, circular adapter ligation, and PacBio sequencing (Figure 5). FLAM‐Seq starts with the selection of a poly(A) + fraction of RNA, which is subsequently subjected to G/I tailing. RNA is reverse transcribed with a primer that consists of a PCR handle on its 5′ end, followed by an N stretch and three T pairing to the added G/I and poly(A) tail, respectively, in the presence of template‐switching oligonucleotide (TSO) for second‐strand synthesis. The created double‐stranded cDNA is then amplified and subjected to PacBio sequencing (Legnini et al., 2019). PAIso‐Seq does not require the selection of polyadenylated RNAs because the 3′ end is extended on a template of oligonucleotides that consist of a TSO sequence minus a triple G on the 5′ end and stretch of T on the 3′ end by Klenow polymerase. Inside the T stretch, two dU nucleotides are present, which enables oligonucleotide degradation with the USER enzyme before RT. Double‐stranded cDNA is synthesized in the presence of primers that correspond to TSO minus triple G and with TSO with triple G, amplified, and subjected to PacBio sequencing (Y. Liu, Nie, et al., 2019). However, the PAIso‐Seq protocol may lead to Klenow‐dependent artifacts because the fill‐in reaction may actually extend the measured poly(A) tails.

The major advantage of all PacBio‐based approaches is the generation of long reads with high accuracy (error rate <0.8%; Legnini et al., 2019), thus enabling isoform‐specific analysis. Moreover, because of PacBio's ability of homopolymer sequencing, it can be used to study the internal composition of poly(A) tails, which was impossible with previous approaches (e.g., TAIL‐Seq allows the detection of only 3′‐end modifications; Legnini et al., 2019; Y. Liu, Nie, et al., 2019). Non‐A nucleotides were also shown to occur within poly(A) tails. However, based on the utilization of T‐A pairing during RT (FLAM‐Seq) or end extension (PAIso‐Seq) to analyze only poly(A) + RNAs, neither method provides data about 3′‐end modifications as TAIL‐Seq does. FLAM‐Seq involves a poly(A) + selection step with oligo(dT) beads that may introduce bias toward RNAs with longer poly(A) tails. This is avoided in PAIso‐Seq, in which polyadenylated RNAs are selected by an end extension template oligonucleotide that contains a T stretch on the 3′ end. Moreover, this improvement increases sensitivity, allowing less starting material with PAIso‐Seq or even performing this protocol with material from a single cell (Y. Liu, Nie, et al., 2019). These features of these two methods are summarized in Table 1.

FLAM‐Seq was used to analyze poly(A) tails in HeLa S3 cells, human induced pluripotent stem cells (iPSCs), iPSC‐derived cerebral organoids, and *C*. *elegans* (L4 larval stage and adult). The obtained poly(A) tails are typically short and correlate well with mTAIL‐Seq data from L4 larval stage *C*. *elegans* (*R* = 0.63; Lima et al., 2017), whereas the correlation of data from HeLa cells that were obtained with TAIL‐Seq (Chang et al., 2014) and PAL‐Seq (Subtelny et al., 2014) is rather low (*R* = 0.03 and *R* = 0.24, respectively; the correlation between TAIL‐Seq and PAL‐Seq: *R* = 0.2; see Table 2). Likewise and as postulated earlier (Lima et al., 2017), in highly expressed genes, poly(A) tails are shorter, whereas median poly(A) tail length correlates slightly negatively with mRNA half‐life and translational efficiency (Legnini et al., 2019). With PAIso‐Seq, mouse germinal vesicle‐stage oocyte poly(A) tails were analyzed, showing a good correlation with TAIL‐Seq data (*Rs* = 0.65; Morgan et al., 2017) and a positive correlation between poly(A) tails and protein abundance in oocytes, which was postulated previously (Lim et al., 2016; Subtelny et al., 2014; Yang et al., 2020; see Table 2).

Another single‐molecule SM‐PAT protocol for poly(A) sequencing with the PacBio platform was recently published (Mattijssen et al., 2020). Total RNA is subjected to the oligo(dT) selection of polyadenylated mRNAs, followed by splint ligation with a 3′ adapter. After RT, the second strand of cDNA is created with random hexamers that are attached to the adapter sequence. After PCR, adapters for SMRT sequencing are added, and the library is loaded onto SMRT Cell V2 and subjected to PacBio sequencing (Figure 5c). This protocol is very similar to FLAM‐Seq and PAIso‐Seq, differing in the 3′‐adapter ligation and cDNA second‐strand synthesis approaches. It was used to analyze the effect of La‐related protein 4 (LARP4) on poly(A) tail length, revealing that this protein promotes the lengthening of poly(A) tails, protects them from deadenylation, and stabilizes mRNAs. Comparisons of the quantification of RNA abundance with this and other methods produced *R* ^*2*^ = 0.58 in the case of RNA‐Seq and a very high *R* ^*2*^ = 0.96 in the case of Northern blot (Mattijssen et al., 2020).

In summary, with the development of third‐generation sequencing technology, the next step in poly(A) tail length studies was made. Long‐read sequencing removes the upper limits of poly(A) tail length detection and gives a more complete view of the mRNA molecule from its 5′ end to 3′ end. However, these methods are still biased by the PCR amplification of poly(A) tails during library preparation. Importantly, PacBio sequencing is quite expensive, and sequencers are not common among laboratories, thus decreasing its versatility and availability.

### 4.4 Nanopore direct RNA sequencing in poly(A) tail length measurement

**FIGURE 6:**
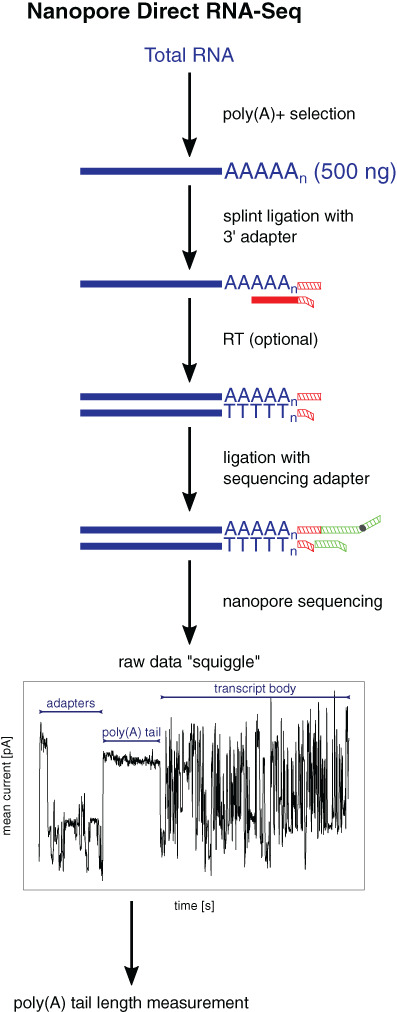
Library preparation workflow for poly(A) tail length measurement with nanopore sequencing and exemplary raw data (squiggle) segmented into adapters, poly(A) tail, and transcript body sequence. The red line indicates the oligo(dT) sequence. Red hatched lines indicate the 3′ adapter sequence (splint adapter). Green hatched lines indicate the sequencing adapter with a motor protein attached (gray circle).

The latest approach for RNA sequencing and poly(A) tail length measurement is direct RNA sequencing (DRS) by ONT (Garalde et al., 2018). This approach is free from cDNA synthesis and PCR amplification, thus enabling the direct, real‐time sequencing of a single RNA molecule and generating full‐length, strand‐specific reads. In addition to standard differential expression analysis, it allows studies of transcript isoforms and splicing (Depledge et al., 2019; Drexler et al., 2020; Roach et al., 2020; Workman et al., 2019; L. Zhao, Zhang, et al., 2019), RNA modifications (H. Liu, Begik, et al., 2019; Lorenz et al., 2020; Parker et al., 2020; Workman et al., 2019), and poly(A) tail length (D. Kim et al., 2020; Krause et al., 2019; Roach et al., 2020; Workman et al., 2019) at the single‐molecule level. The sequencing library workflow (Figure 6) consists of splint ligation of the 3′ adapter to a polyadenylated RNA molecule, followed by the optional RT and ligation of a sequencing adapter that is attached to a motor protein. The library is loaded on a flow cell that is created by protein pores (nanopores) that are embedded in an array of synthetic membranes. After applying the voltage, RNA passes through the nanopore at a constant speed, driven by motor protein, in a 3′–5′ direction, creating a specific perturbation of current that is converted into a base sequence using a trained neural network algorithm (Garalde et al., 2018; Stark et al., 2019). Importantly, even if the cDNA strand is synthesized, only RNA is sequenced because of the motor protein that is attached to the sequencing adapter.

Compared with the previously described methods, the determination of poly(A) tail lengths is more challenging because standard base‐calling tools are unable to call long homopolymers correctly. Thus, poly(A) tail lengths cannot be determined directly from sequence files. Therefore, a specific data analysis pipeline based on the raw current intensity signal is required using Nanopolish‐polyA (https://github.com/jts/nanopolish; Workman et al., 2019) or TailfindR (https://github.com/adnaniazi/tailfindr; Krause et al., 2019). Both rely on proper segmentation of the raw current signal into a sequencing adapter, splint adapter, poly(A) tail, and eventually transcript body (Figure 6) but using different approaches. Nanopolish‐polyA uses the hidden Markov model to find segment boundaries, whereas TailfindR looks for regions in the normalized current intensity signals with defined values. Having boundaries of poly(A) in the raw signal, the time it takes for the poly(A) tail to pass through a pore can be calculated. Additionally, based purely on the raw signal (for TailfindR) or alignment of the raw signal to the base‐called and reference‐mapped sequence (for Nanopolish‐polyA), the speed at which the RNA passes through the pore can be calculated (deviations from the expected ~70 nt/s are observed). Finally, by determining the dwell time of the poly(A) region in the pore and the average translocation speed, the length of the poly(A) tail can be calculated using the following formula: poly(A) length = poly(A) dwell time × average translocation speed.

The algorithm that is implemented in Nanopolish‐polyA was tested on sequencing data from a human B cell lymphoma cell line, obtaining an average poly(A) size of 50 nt, which has been reported previously (Chang et al., 2014; Subtelny et al., 2014), and mean poly(A) tail length of 112 nt for nuclear transcripts. The poly(A) tail length can also be transcript isoform‐specific (Workman et al., 2019) and its effect on RNA stability can be assessed for each isoform (Maier et al., 2020). This approach was also used in studies of *C*. *elegans* (Roach et al., 2020), showing similar poly(A) tail length distributions as previously published data from mTAIL‐Seq (Lima et al., 2017) in the L4 stage. Moreover, in the larval stage, poly(A) tails are shorter than in adults and correlate negatively with gene expression, which is lost during development (Roach et al., 2020). Analyses of direct RNA‐Seq data with Nanopolish‐polyA were also performed to estimate poly(A) tail lengths in studies of *A*. *thaliana* (Parker et al., 2020), splicing in human K562 cell RNA with or without in vitro polyadenylation (Drexler et al., 2020), and cytoplasmic polyadenylation by TENT5C in mouse B cells (Bilska et al., 2020) and TENT5A in osteoblasts (Gewartowska et al., 2021) and to show poly(A) tail shortening in human adenovirus type 5 with the progression of infection (Donovan‐Banfield et al., 2020). It was also used to study the metabolism of poly(A) tails in the yeast *Saccharomyces cerevisiae* (Tudek et al., 2021; Turtola et al., 2021).

TailfindR is less widely used than Nanopolish‐polyA, despite the fact that the correlation of results that are computed with these two methods is good (*R* = 0.98 for poly[A] tail lengths) (Krause et al., 2019). The discrepancies are probably caused by different approaches to calculating poly(A) boundaries. Both tools were also compared in poly(A) tail analyses of human HAP1 and HEK293 cell lines, giving comparable results with a modal poly(A) tail length that was similar to the one that was measured with TAIL‐Seq (Chang et al., 2014; Soneson et al., 2019). TailfindR also has improvements in data analysis based on ONT cDNA sequencing, measuring both poly(A) and poly(T), which reduces the amount of input material and increases the number of reads. Moreover, the cDNA approach generated tail lengths even more precisely than RNA (Krause et al., 2019). Recently, Nanopore 3 Prime end‐capture sequencing (Nano3P‐Seq), another approach for poly(A) tail length profiling, was developed, relying on direct cDNA nanopore sequencing (Begik et al., 2021). In this protocol, the template‐switching reverse transcriptase TGIRT is used to capture all, even non‐polyadenylated, RNA species, and TailfindR is used to calculate poly(A) tail lengths. This method was used to study polyadenylation dynamics during the maternal‐to‐zygotic transition at the isoform‐specific level (Begik et al., 2021). Interestingly, the authors of Nano3P‐Seq noticed a consistent bias of 15 nt in TailfindR‐calculated poly(A) lengths compared with poly(A) standards, thus obtaining results that required postprocessing.

Any long homopolymer at the 3′ end of RNA can be detected with DRS. This aspect was used by the authors of Nanopolish‐detect‐polyI and employed in the nano‐COP protocol (Drexler et al., 2021). Nano‐COP aims to analyze co‐transcriptional processing by Nanopore sequencing. Because nascent RNA is not fully polyadenylated, RNA must be enzymatically tailed to perform sequencing. However, this approach makes it impossible to distinguish poly(A) tails of mature RNA from enzymatically added poly(A) tails. To overcome this issue, RNA can be tailed by poly(I), which allows the preparation of libraries using an appropriately designed adapter. Nanopolish‐detect‐polyI can then distinguish between poly(I) and poly(A) tails and measure the length of poly(A) tails (Drexler et al., 2021).

Recently, poly(A) tails of SARS‐CoV‐2 RNAs were measured with a not yet published poreplex tool (https://github.com/hyeshik/poreplex), showing median poly(A) tail lengths of 47 nt and a difference in poly(A) tail length between modified and unmodified RNAs (D. Kim et al., 2020). The authors utilized a similar approach as Nanopolish and TailfindR to measure the dwell time of the poly(A) tail. The dwell time was converted to a length by normalization to 1/30 of the dwell time of the 30‐nt poly(A) tail from the ONT calibration strand that was added to the library (D. Kim et al., 2020). Thus, the normalization method is the main difference between these tools.

Direct RNA sequencing is still a novel method, and subsequent data analysis tools are still being developed. Computational pipelines allow the simultaneous analysis of various aspects of RNA biology. One example is the MasterOfPores (Cozzuto et al., 2020) pipeline for direct RNA‐Seq data analysis. One of its modules, NanoTail, performs poly(A) tail length estimations with Nanopolish and TailfindR algorithms to generate data separately from both methods as well as computing correlations between the data that are obtained with them (Cozzuto et al., 2020). For the visualization and analysis of Oxford Nanopore direct RNA‐Seq‐based poly(A) predictions, the R package NanoTail (https://github.com/pbrigf-ibb/nanotail) was created, but it has not yet been published. It allows statistical comparisons of poly(A) lengths between multiple datasets and was used in several published works that employed DRS for poly(A) analysis (Bilska et al., 2020; Gewartowska et al., 2021).

Additionally, information about poly(A) tail length can be obtained from the sequencing of cDNA after amplification as shown in FLEP‐Seq (Long et al., 2021). This protocol is used to study nascent transcripts by utilizing adapter ligation to capture both poly‐ and non‐adenylated mRNAs, followed by cDNA synthesis, amplification, and nanopore sequencing. However, it requires dedicated PolyAcaller (Long et al., 2021) software to measure poly(A) tails as Nanopolish can be used only for RNA, and Tailfinder that has been designed for cDNA generates high false‐positive rates. The developed protocol can also be used for PacBio sequencing. Poly(A) tails that are measured with both approaches show a good correlation (*R* = 0.89; Long et al., 2021).

Apart from the many advantages of Nanopore sequencing, its major disadvantages are the high error rate (Rang et al., 2018) and the requirement for a relatively large amount of starting material (500 ng of poly[A] + RNA) for direct RNA‐Seq, which may be biased toward RNAs with longer poly(A) tails, depending on the poly(A) selection method used (Ibrahim et al., 2021; Tudek et al., 2021). Similar to mTAIL‐Seq and PAL‐Seq, because of the utilization of splint ligation to the 3′ adapter, it captures only polyadenylated RNAs, and a modified protocol is needed to study RNAs with non‐poly(A) 3′ ends. Table 1 shows the major features of this method compared with other previously described methods.

Direct Nanopore sequencing was the first PCR‐free method to study poly(A) tail length, representing the latest accomplishment in the development of these methods. The estimation of poly(A) tail length that is not limited by reading length and not biased by PCR amplification seems to be a solution to problems with the previous techniques. Additionally, the protocol for library preparation is short and easy to perform. The required equipment includes a portable and relatively inexpensive sequencer that is connected to a laptop computer, making this protocol easy to implement in most laboratories, resulting in the rising popularity of nanopore sequencing for poly(A) tail length measurements. Importantly, no special protocol for poly(A) tail analyses is needed, meaning that we can perform various analyses in one sequencing run, including differential expression, splicing isoforms, modifications, and co‐transcriptional processing. Although there are still limitations of this approach, ONT is constantly improving the sequencing equipment and chemistry. Users are generating new versions of protocols for library preparation and computational analyses, making this technique very promising.

## 5 CONCLUSION

Tailing of the 3′ end of mRNA is an important post‐transcriptional modification that contributes significantly to the regulation of the expression of genetic information. Therefore, it requires an appropriate methodology to study it properly. During the last 50 years, we have witnessed fascinating developments in the methods that are used to study poly(A) tail length, starting with RNaseH‐ and PCR‐based approaches, which opened this field of research. Their significant limitation with regard to throughput was overcome by the development of microarray technology and studying transcripts that are fractionated by their poly(A) tail length. However, none of these methods provided information about exact poly(A) tail lengths or possible modifications. A revolution occurred with the development of NGS. The first adjustments in sequencing protocols were made to enable studies of poly(A) tail length. Publications that were based on TAIL‐Seq (Chang et al., 2014) and PAL‐Seq (Subtelny et al., 2014) enhanced our knowledge of the 3′ terminome, providing more accurate, high‐throughput data on poly(A) tail length and the detection of its modifications. Despite their undeniable impact on the field, these methods are characterized by the unavoidable bias of PCR amplification of long homopolymers, poly(A) tails of which lead to underestimations of poly(A) tail length, and notable technical challenges. Additionally, the measured lengths of poly(A) tails are limited to read length, leading to the loss of information about very long tails. These problems can be overcome by measuring poly(A) tail lengths using single‐molecule third‐generation sequencing. Protocols that are based on the PacBio platform, such as FLAM‐Seq and PAIso‐Seq, still introduce the PCR amplification of libraries. Oxford Nanopore Technologies offers a direct RNA or direct cDNA sequencing approach, leading to non‐PCR‐biased poly(A) tail length estimations. Because it generates long reads compared with short‐read NGS, poly(A) tail measurements can be accompanied by analyses of other aspects of RNA biology, such as splicing isoforms and base modifications.

In conclusion, the massive area of 3′ terminome research requires more exploration. Further developments in poly(A) tail sequencing still need to be made. The utilization of Nanopore sequencing is gaining popularity, and we predict further improvements in protocols based on this technology.

## AUTHOR CONTRIBUTIONS

**Aleksandra Brouze:** Conceptualization (equal); funding acquisition (equal); visualization (lead); writing – original draft (lead); writing – review and editing (lead). **Paweł Szczepan Krawczyk:** Writing – original draft (equal). **Andrzej Dziembowski:** Conceptualization (supporting); supervision (supporting); writing – original draft (supporting). **Seweryn Mroczek:** Conceptualization (equal); funding acquisition (equal); supervision (lead); writing – original draft (equal).

## FUNDING INFORMATION

This work was supported by the National Science Centre (Preludium‐19 UMO‐2020/37/N/NZ2/02893 to Aleksandra Brouze and Sonata Bis 10 UMO‐2020/38/E/NZ2/00372 to Seweryn Mroczek) and by the Foundation for Polish Science (FNP) (to Aleksandra Brouze).

## CONFLICT OF INTEREST

The authors have declared no conflicts of interest for this article.

## RELATED WIREs ARTICLES

Polyadenylation and beyond: Emerging roles for noncanonical poly(A) polymerases

Functions and mechanisms of RNA tailing by metazoan terminal nucleotidyltransferases

Control of poly(A) tail length

## Data Availability

Data sharing is not applicable to this article as no new data were created or analyzed in this study.
